# Tutorial on In Situ and *Operando* (Scanning) Transmission Electron Microscopy for Analysis of Nanoscale Structure–Property Relationships

**DOI:** 10.1021/acsnano.4c09256

**Published:** 2024-12-18

**Authors:** Michelle A. Smeaton, Patricia Abellan, Steven R. Spurgeon, Raymond R. Unocic, Katherine L. Jungjohann

**Affiliations:** † National Renewable Energy Laboratory, Golden, Colorado 80401, United States; ‡ Nantes Université, CNRS, Institut des Matériaux de Nantes Jean Rouxel, IMN, F-44000 Nantes, France; ¶ National Renewable Energy Laboratory, Golden, Colorado 80401, United States; ∇ Renewable and Sustainable Energy Institute, University of Colorado Boulder, Boulder, Colorado 80309, United States; § Oak Ridge National Laboratory, Oak Ridge, Tennessee 37831, United States

**Keywords:** nanoscale structure−property relationships, in situ, *operando*, (S)TEM, tutorial

## Abstract

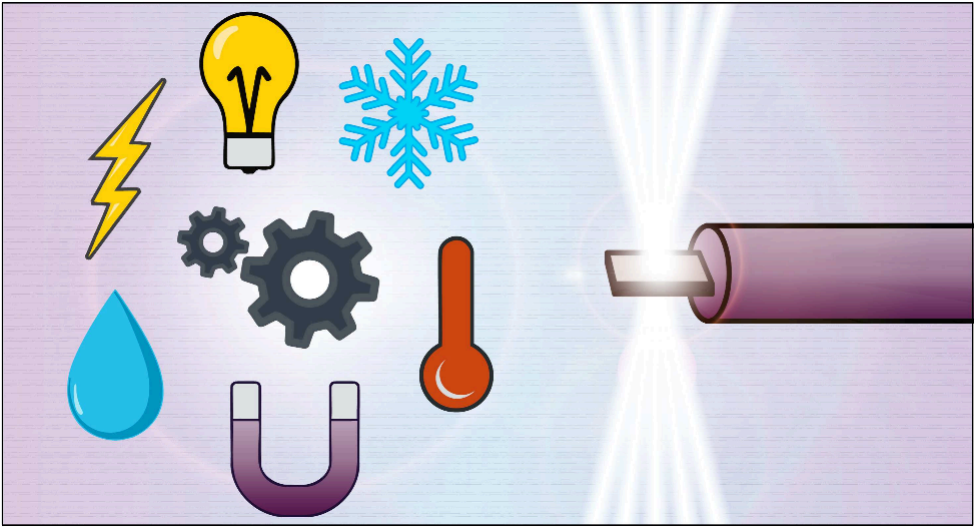

In situ and *operando* (scanning) transmission electron microscopy [(S)TEM] is a powerful characterization technique that uses imaging, diffraction, and spectroscopy to gain nano-to-atomic scale insights into the structure–property relationships in materials. This technique is both customizable and complex because many factors impact the ability to collect structural, compositional, and bonding information from a sample during environmental exposure or under application of an external stimulus. In the past two decades, in situ and *operando* (S)TEM methods have diversified and grown to encompass additional capabilities, higher degrees of precision, dynamic tracking abilities, enhanced reproducibility, and improved analytical tools. Much of this growth has been shared through the community and within commercialized products that enable rapid adoption and training in this approach. This tutorial aims to serve as a guide for students, collaborators, and nonspecialists to learn the important factors that impact the success of in situ and *operando* (S)TEM experiments and assess the value of the results obtained. As this is not a step-by-step guide, readers are encouraged to seek out the many comprehensive resources available for gaining a deeper understanding of in situ and *operando* (S)TEM methods, property measurements, data acquisition, reproducibility, and data analytics.

## Introduction

**Figure 1:**
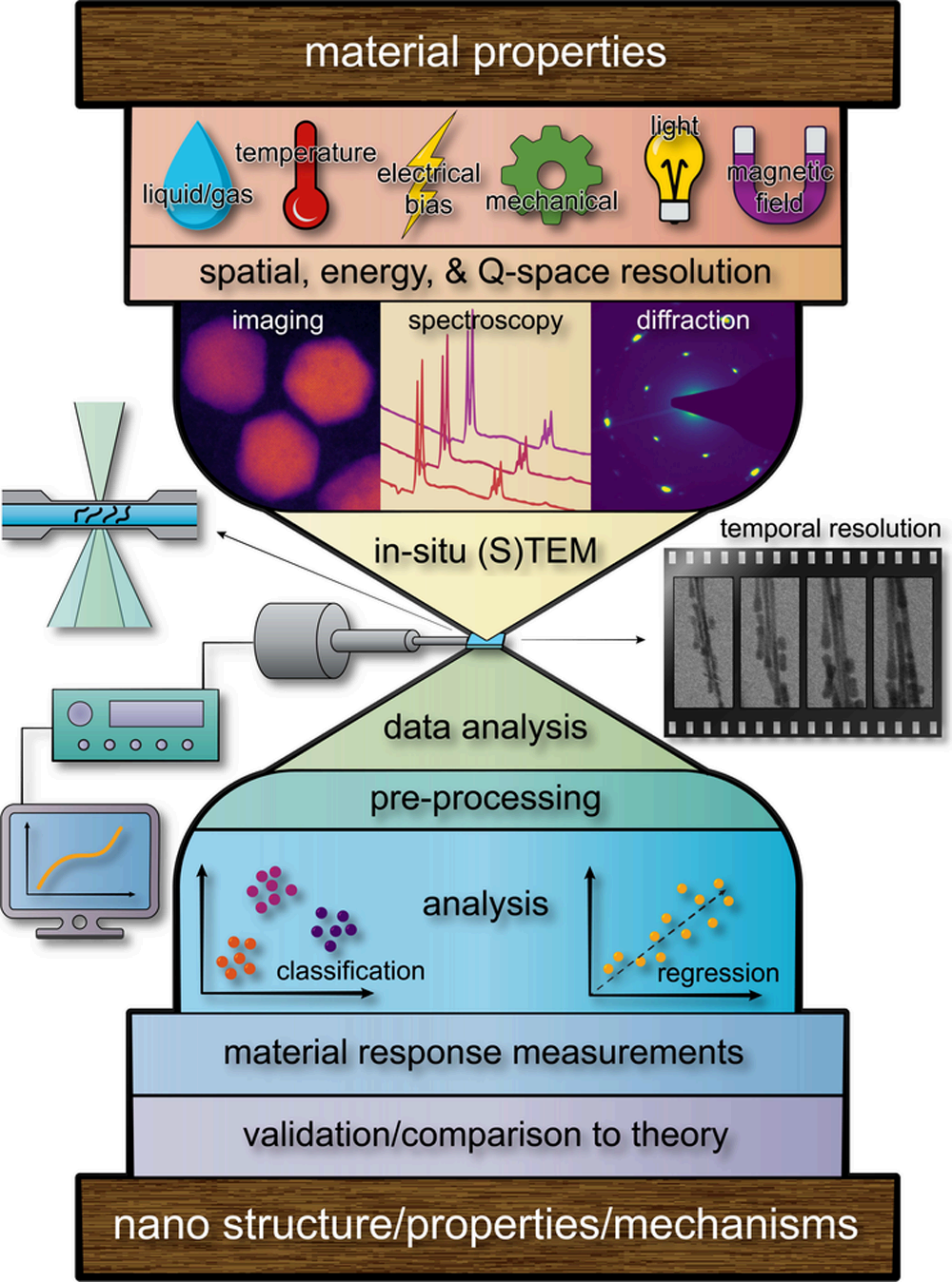
Overview of the in situ (S)TEM workflow and its application for understanding nano-to-atomic scale material properties, feature growth, chemical reactions, phase transformations, defect dynamics, and transient states. The experimental workflow comprises: selecting and applying relevant stimuli (including external biases and sample environments); acquiring time-dependent imaging, diffraction, and/or spectroscopy data; analyzing the data to extract nanoscale properties and mechanisms; and subsequently comparing the conclusions to bulk measurements and/or theoretical models. (Note that Q-space refers to reciprocal/diffraction space.).

In situ and *operando* (scanning) transmission and transmission electron microscopy [(S)TEM] offers a powerful platform for investigating materials behavior under a variety of stimuli and environmental/device conditions.^1−6^ It enables nanoscale spatial resolution, eV-to-meV energy resolution, and adaptable temporal resolution, allowing researchers to observe the structural and chemical evolution of key material features, including grains, interfaces, surfaces, and defects. Figure 1 shows an overview of a general in situ or *operando* workflow: selecting and/or combining relevant stimuli (including external biases and sample environments); collecting time-dependent imaging, diffraction, and/or spectroscopy data; analyzing the data to extract nanoscale properties and mechanisms; and then validating the results against bulk measurements and/or theory.

With the use of aberration correction, (S)TEM imaging techniques can now routinely reach spatial resolutions below 1 Å,^7,8^ while associated spectroscopic techniques are routinely performed at the atomic scale, including energy-dispersive X-ray spectroscopy (EDS) and electron energy loss spectroscopy (EELS).^9−11^ This spatial resolution means (S)TEM techniques can provide site specificity for in situ measurements that is inaccessible to other structural and spectroscopic characterization techniques, including X-ray and neutron scattering, Raman spectroscopy, Fourier transform infrared (FTIR) spectroscopy, and X-ray photoelectron spectroscopy (XPS). Furthermore, a variety of sample preparation techniques are available to enable the analysis of a wide range of sample geometries and features (see Sample and Measurement Design subsection). For example, focused ion beam (FIB) lift-out enables direct characterization of buried interfaces and structures in heterogeneous systems and devices. (S)TEM enables unrivaled flexibility in collecting a range of data from many sample geometries, which can be custom-prepared to extract nanoscale structure–property relationships at a selected location in a sample.

Modern electron sources can also produce beams with energy spreads on the order of 1 eV, enabling elemental mapping and quantification (EDS and EELS) and analysis of valence states and bonding environments (EELS near-edge structure analysis). Monochromation can improve the inherent resolution significantly, down to a few meV in some cases, which approaches energy resolutions available with synchrotron X-ray absorption spectroscopy (XAS).^12,13^

The achievable time resolution in an in situ (S)TEM experiment is highly dependent on the type of data being collected (imaging, diffraction, and/or spectroscopy), the choice of illumination mode (STEM or TEM), and the detector being used.^14,15^ Cutting-edge detectors can record hundreds of frames per second in a standard (S)TEM, with even higher speeds available in specialized ultrafast TEM instruments,^16−18^ which capture so many images/spectra that the generated (terabyte scale) data must be analyzed with high-throughput methods and robust computers (see the Data Analytics section).

The utility of the (S)TEM can be further expanded by subjecting the sample to desired conditions. A plethora of side-entry (S)TEM holders have been designed to apply various stimuli to samples in the microscope column (see Physical Property Measurements section). The sample environment can be altered by introducing liquids and gases in static^19−22^ or flow cell holders;^23−25^ the sample temperature can be elevated^26^ or decreased from room temperature;^27^ the sample can also be subjected to controlled electrical biases,^19^ magnetic biases,^28^ and mechanical forces^29^–all while the (S)TEM records the resulting material responses. Some (S)TEM instruments can even incorporate stimuli into the microscope column itself. For example, environmental (S)TEMs can flow low-pressure gases into the column, and integrated ion and laser sources can be used to irradiate or photoexcite the sample.

Experiments in which one or more stimuli are applied to a sample while data is collected are typically identified as either “in situ” or “*operando*,” but it is useful to clarify the differences between these terms. “*Operando*” measurements assess a sample’s response and evolution under its intended operating conditions; however, true *operando* conditions are difficult to achieve in (S)TEM experiments, due to limitations on sample size and thickness and the need for high vacuum to maintain the electron optics quality. In situ more generally refers to the characterization of a sample under an applied stimulus or environment, which may mimic a particular point in materials synthesis or device operation but lack the complexity of the bulk or native working conditions. To validate the relevance of the in situ observations for specific synthesis or device conditions, in situ characterization is often paired with analogous ex situ and/or bulk measurements.

In short, in situ and *operando* (S)TEM experiments are highly complex, and immense care must be taken in designing experiments to extract the desired information. From the outset, it is important to consider: (1) what type of data will be required (imaging, diffraction, and/or spectroscopy), (2) how the sample will be prepared, (3) how the beam may interact with the sample, (4) how the beam-sample interaction may be affected (or even enhanced) by the applied stimulus, (5) how reproducible the results may be, (6) whether the results are representative, and (7) how the data will be analyzed and interpreted. It is generally possible to optimize the sample and experimental conditions for accurate nanoscale property measurements with high spatial or temporal resolution, but there are many trade-offs to be considered (e.g., speed of data acquisition, signal-to-noise ratio of the data, and sample specifications). This tutorial is divided into three sections that expand on the most important considerations for collecting and interpreting in situ and *operando* (S)TEM data: “Physical Property Measurements” will address sample measurement and design and experiment execution; “Data Acquisition: Defining Beam Parameters and Avoiding Artifacts” will cover selection of imaging mode and beam effects on specimens and property measurements; “Data Analytics: Setting Analysis in Motion” will cover methods for data analysis and discuss the opportunities and limitations of machine learning and artificial intelligence for this task. More detailed resources are available for readers interested in gaining a deeper understanding of in situ and *operando* (S)TEM techniques and experiment design.^30−32^

## Physical Property Measurements

In designing an in situ (S)TEM experiment to capture a desired material response, it is wise to let your desired output data drive the choice of the applied stimuli and environment (e.g., liquid, gas, heat, bias, magnetic field), the sample preparation technique (e.g., drop casting, FIB lift out, nanomanipulation), and the data collection modality (imaging, diffraction and/or spectroscopy).

**Figure 2:**
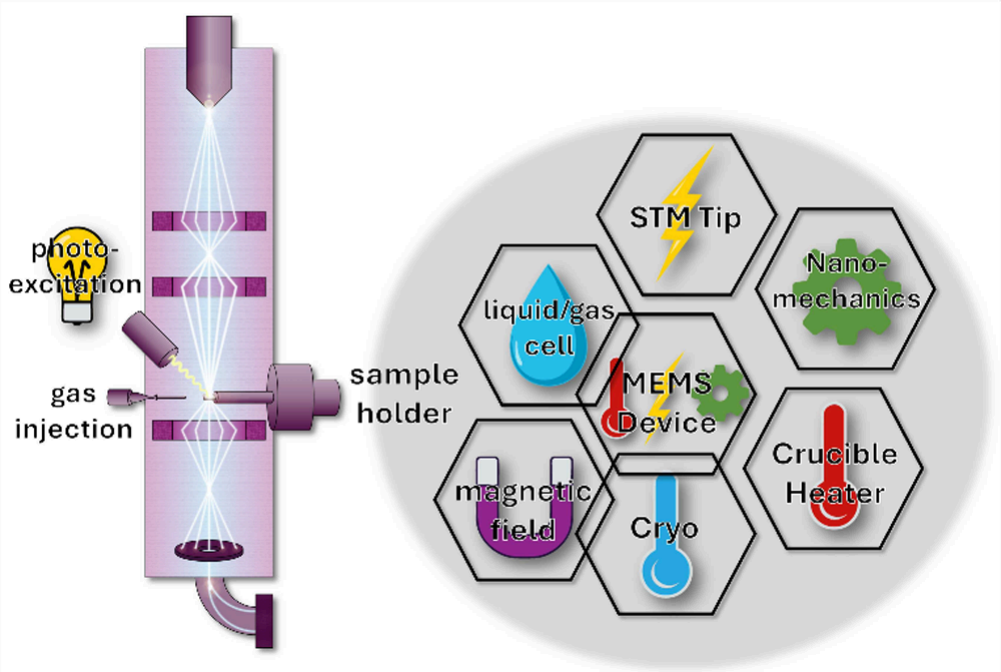
Schematic of in situ environments and external stimuli available for in situ holders and microscopes. Tiles represent holder types, and colored icons represent stimuli enabled by those holders. Note that MEMS (micro electro mechanical system) devices can be designed to produce multiple different stimuli, individually or simultaneously. Overlapping tiles indicate routine availability of holders with combined functionalities.

Recognizing the value of in situ and *operando* experiments, microscope and TEM holder companies now offer a wide range of holders and microscope column additions for applying stimuli and environmental conditions; many researchers have also chosen to build or modify holders to fit their specific experiments. Figure 2 depicts the common external stimuli available for in situ and *operando* holders/microscopes;^19,24,33−42^ connections between the tiles indicate the routine availability of TEM holders with combined functionalities.

When scaling an in situ or *operando* experiment down to the micro-to-nano scale, it is important to remember that the conditions needed to create bulk reactions may not be identical to those needed for nanoscale reactions. For instance, a highly corrosive solution used in bulk experiments may initiate a nanoscale reaction even before the sample and holder are inserted into the microscope. In that case, a less corrosive solution may better reveal the initial nanoscale reactions in the (S)TEM, even if that solution is a less precise match to “real” stimulating conditions.^43^

It may seem desirable to conduct every experiment under *operando* conditions, because great value can be obtained by collecting data from a material reacting in its native or working environment. Unfortunately, complex native environments are often extremely difficult to mimic within the high vacuum of an electron microscope because most native environments have a complicated combination of stimuli, some of which may be “unknown unknowns,” including natural contaminants or ion mobility from interfaces far away from the region of study. For example, to simulate the microenvironment of a nanometric interface/structure well enough to replicate a damage mechanism or less-common reaction, a robust baseline understanding must first be developed, often through ex situ measurements taken to define and prioritize the conditions (temperature, gas chemistry, gas pressure, liquid chemistry, humidity, contaminants, bias, stress, and magnetic fields) that are most desirable (and feasible) for (S)TEM imaging.^19,24,33−42^

### Sample and Measurement Design

**Figure 3:**
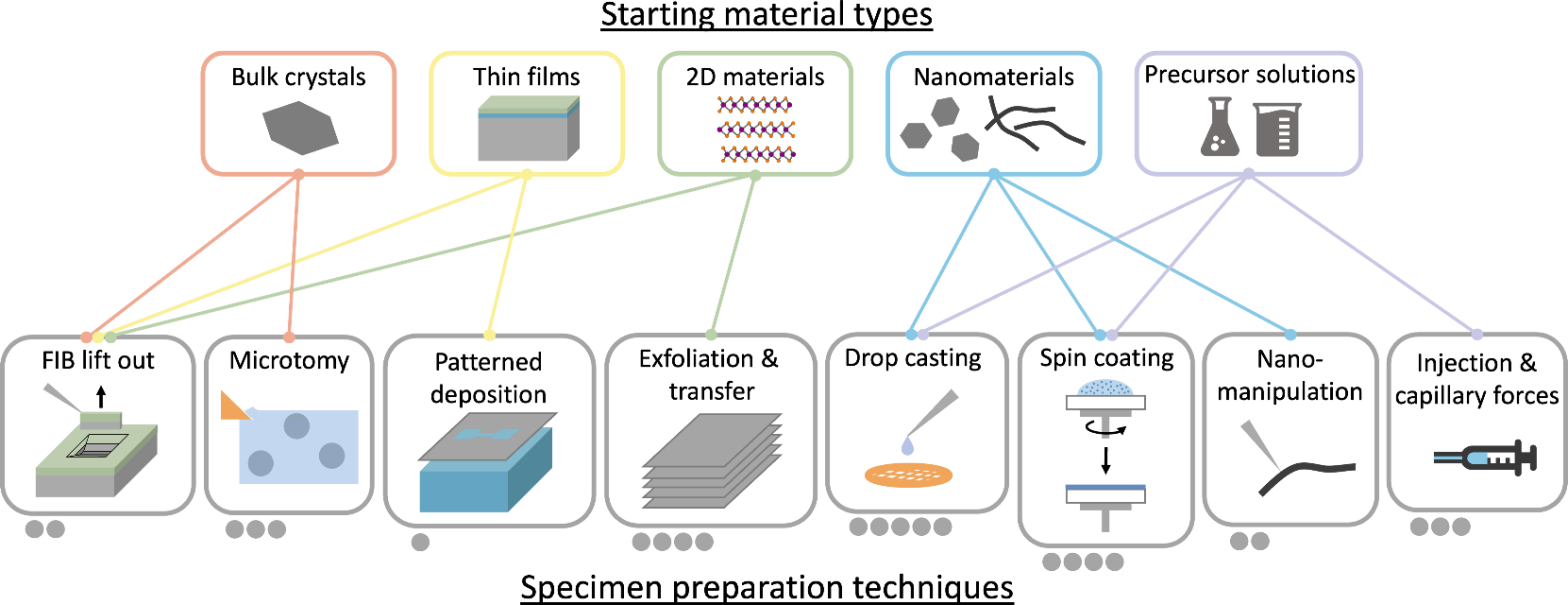
Diagram of starting material classes and commonly used (S)TEM specimen preparation techniques, including manipulation and transfer methods. Each preparation technique is connected to the most compatible starting sample type(s). An ease-of-use rating is represented by the gray circles, from drop casting (simplest) to microfabrication (most onerous).

Once the environment/stimulus has been selected, the next step is to select the optimal specimen geometry for the in situ or *operando* experiment. A variety of standard starting sample types are regularly prepared for (S)TEM analysis (Figure 3), following a range of different procedures too broad for discussion here. Standard specimen preparation, modification, and transfer techniques can be used to produce specimens compatible with in situ or *operando* (S)TEM measurements. The choice of starting material and a (S)TEM specimen preparation technique often hinges on the sample source provided, equipment availability, and compatibility of the available starting materials with physical property measurements. Unfortunately, it is not always possible to access all desired material synthesis resources or purchase custom-designed samples.

#### Accessing Specifically Defined Sites and Locations

Sometimes, the question being studied requires access to specifically defined sites (e.g., grain boundaries and interfaces), making it necessary to employ a site-specific specimen preparation technique (e.g., FIB-lift out) or use a starting material designed to contain the features of interest in the area being probed (e.g., a solution of twinned nanoparticles). For example, consider the task of investigating a material coating to determine whether it reduces the surface reactivity of the substrate. In this case, many different starting materials could be used to access the coating/substrate interface, including a bulk crystal, a thin film, nanocrystals, or nanowires of a metal with a coating deposited on the already prepared (S)TEM specimen; or a thin film, where the coating was prepared prior to (S)TEM specimen preparation. This decision could be made based on availability of starting materials and TEM holders, desired specimen thickness, and/or correspondence to bulk application of interest.

#### Effects of Specimen Holder and Chip Designs

**Figure 4:**
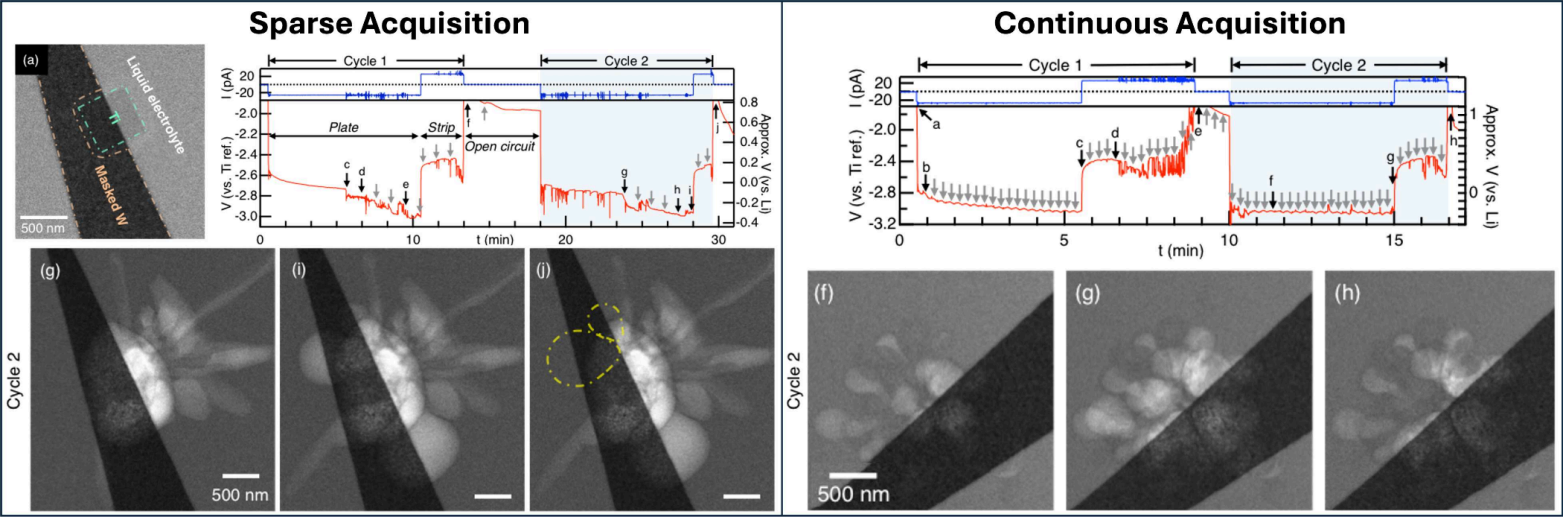
First two plating and stripping cycles of lithium electrodeposition captured using sparse and continuous (every 15 s) acquisition of bright-field STEM images during galvanostatic application of ±20 pA to measure the voltage profile of a 0.26 μm^2^ titanium electrode in carbonate electrolyte. Adapted with permission from Leenheer et al.^22^ Copyright 2015 American Chemical Society.

The choice of the ideal starting sample type and specimen preparation technique also depends on the available options for holder and specimen support components (e.g., grid, electron-transparent window, micro electro mechanical system (MEMS) device/chip, electrode, holder). Few electron microscopes have identical configurations or compatible holders, which means that there is necessarily wide variance in sample attachment/placement/location and, thus, wide variance in experimental design. Attention to detail is imperative because sample attachment/placement/location impacts both the imaging conditions and property measurements. For example, if the sample for a biasing experiment is electrodeposited on a planar electrode, the resulting characterization will include unwanted background imaging signal (from the electrode material) and biasing signal (from the electron beam), as shown in Figure 4. Furthermore, because the electrode increases the specimen thickness, it will impact both the quality of the images (thicker specimens reduce imaging contrast) and spectra (thick samples cause multiple scattering of the imaging electrons). We note that thin specimens are critical for some techniques (e.g., EELS) but less essential for others (e.g., EDS).

#### Measuring the Correct Specimen Features

How do we design the experiment to ensure (to the best of our ability) that the property we are measuring is from the sample and not from the background/contacts or an interaction between the sample and background/contacts? Consider the case of a FIB/SEM biasing or electrical measurement: the relative resistance between the sample and the contacts made in the FIB/SEM is important because the contact resistance will vary based on the material (Pt, C, or W) and curing beam (ion or electron).^44,45^ For example, an electrochemical lithiation experiment that uses ion-beam-deposited platinum contacts to enable lithium insertion into the material may result in the lithium being inadvertently inserted into the platinum contact rather than the material of interest. Similarly, contact characteristics are important in mechanical property measurements because contacts that are too compliant may deform before the sample, leading to an incorrect property measurement. To rule out the introduction of artifacts in the property measurements, the chemical compatibility and property measurements of all connections to the sample material should be carefully evaluated.

As you choose your starting material and specimen preparation technique, consider the following questions: *(1) What part of the specimen will the imaging/diffraction/spectroscopy explore* (e.g., interface or bulk, in-plane or cross-sectional orientation)? Pristine materials gown/deposited in the ideal configuration will provide artifact-free results, but ideal specimen preparation is not always an option. Property measurements must account for nonideal specimen preparation; for example, many samples are thinned via FIB, even though this process can implant Ga-ions into the specimen and risks Ga-ion segregation (e.g., at grain boundaries or along surfaces), which might alter the mechanical, thermal, electrical, or electrochemical properties of the specimen. *(2) How will the directionality of the stimulus or environment affect the sample?* For instance, in a liquid or gas, will there be directional flow; if so, how will the sample be positioned relative to this flow? Similarly, for biasing and mechanical straining experiments: which direction will activate/direct the current or strain pathway through the region of interest *(3)What is the optimal specimen thickness for the desired (S)TEM modality?* Sometimes the optimal specimen thickness depends on the desired image resolution/quality or spectral signal-to-background ratio. For example, atomic-scale imaging and high-energy-resolution EELS usually require extremely thin (≤30 nm) specimens, while high-signal EDS or trapped strain measurements can benefit from thicker (150–250 nm) specimens.

As you assess the positive and negative impacts of various sample preparation methods, look up publications for similar experiments and take guidance from the Supporting Information, where researchers often provide the detailed reasoning for their experimental design. User facilities that provide free access to instrumentation and experimental design expertise are also valuable resources that can save you troubleshooting time and expense.

### Performing the Measurement

#### Loading the Specimen

Once the experiment is planned and the specimen is prepared, the specimen must be correctly loaded into the holder (Figure 2), and the holder loaded into the (S)TEM. Published video articles detail these methods, with consideration for different stimuli^34,46−49^ that are designed to prevent unintended reactions or modifications to the specimen prior to the initiation of the in situ or *operando* experiment. For example, imagine loading a MEMS chip into a biasing holder. Once the holder is loaded into the pole piece of the (S)TEM, it may make electrical contact with the internal −2 V bias of the goniometer; grounding the holder during loading reduces the concern that this contact will impact the specimen prior to observation. A very delicate specimen (e.g., a nanoscale resistive random access memory interface) could also be damaged by exposure to static discharge in the laboratory (easily incurred by friction during holder handling or loading), which would compromise the specimen’s pristine state or even completely disfigure the specimen. Handling is also a concern for nanomechanics holders because any jarring motion could impact the specimen or the orientation of the sensor, causing the experiment to fail. Liquid cells are challenging because the benign solutions/solvents sometimes used to enable controlled initiation of the reaction conditions must be displaced by a reactive solution/solvent, and the fluid flow could damage or dislodge a specimen. Alternatively, once a static environmental cell (e.g., an epoxy sealed MEMS chip or graphene cell) is sealed, the environment cannot be altered, so researchers generally try to image the static-cell specimens immediately after sealing (though loading the holder can still take 10–20 min). In short, the loading process is crucial to ensuring the effective execution of in situ and *operando* (S)TEM studies.

#### Capturing the Process

Once the sample holder is loaded in the (S)TEM, an initial state image/diffraction pattern/spectrum should be collected from the region of interest. It is important to identify the correct specimen region for data acquisition to produce results that correlate to the property measurement. The goal is to probe the process responsible for the physical property measurement you are simultaneously acquiring and not to “miss it”. For example, when collecting mechanical strain data from a metal bar, if the imaging is performed near atomic scale, the mechanical failure may not be captured (unless the metal bar is very small) because the failure may occur at any point along the bar and could, therefore, easily occur outside the viewing area. In this specific case, some researchers use a notch in bar specimens to initiate fracture at a specific site.^50^ In some cases, finding the region of interest and obtaining an initial-state image, diffraction, or spectrum is also challenging because it requires exposing fragile specimens to irradiation by the incident electron beam before the experiment begins. For example, in samples that are highly sensitive to the electron beam (e.g., most liquid experiments) collecting initial-state data is extremely challenging because beam-induced processes can dominate or alter the chemical environment around the specimen. In these cases, researchers tend to favor low electron-dose conditions and (if possible) image at unexposed specimen sites throughout the experiment. The beam tolerance of the materials under the given environmental conditions will dictate the amount of pre-experiment data that can be acquired without compromising the specimen’s structural and compositional integrity.

#### Performing Property Measurements

Executing property measurements during *operando* or in situ experiments requires careful calibration and monitoring of the measurements while the images/diffraction patterns/spectra are being collected. Therefore, it is important to record background levels for the property measurements (e.g., indicating the resistance through the connections or measurement frequency and range of motion values for straining experiments) either before or (in some cases) after the in situ experiment.^51^ During the experiment, careful attention to the data collection from the microscope and property measurements will ensure that the timestamps and event monitoring can be correlated between the different data streams, which will aid in identifying correlations between the structural/compositional changes of the specimen and the events measured for the material’s response. Advanced software packages available for some in situ holders can provide this level of data correlation and processing without manual alignment.^52^

To achieve a comprehensive understanding of the in situ or *operando* experimental data, the entire process (from specimen preparation, to experiment design, to implementation) requires meticulous care to avoid or account for variability and unintended interactions. The past two decades of in situ (S)TEM holder, software, and methods development have produced many streamlined tools and techniques for rapid analysis and reproducible data collection.^53−55^ Taking advantage of these learned practices and tools will well-situate researchers starting out with in situ experiments to aid in the creation and development of even more advanced systems, a welcome contribution to the in situ and *operando* (S)TEM field.

Section Summary:

- Experimental design should cater to the material, stimuli, and targeted science question.
- Scaling the experiment to nanometric features and choosing specific stimulating conditions requires a good baseline understanding of the process of interest (often developed through ex situ experiments).
- It is important to minimize impact to the specimen’s structure/properties as the specimen is mounted on the holder, the holder is loaded into the electron microscope, and initial-state data is obtained.

## Data Acquisition: Defining Beam Parameters and Avoiding Artifacts

(S)TEM instruments produce a range of imaging, diffraction, and spectroscopic signals that can be collected during an experiment (often simultaneously) at a wide range of spatial and energy resolutions, making in situ (S)TEM studies a powerful method for understanding nanoscale properties and dynamics. However, these signals have different requirements regarding electron dose and data collection speed, specimen thickness, etc. These requirements then have consequences for the types of dynamics that can be captured during in situ measurements. To optimize electron beam parameters and collected data streams, it is necessary to consider the specimen’s geometry, stimulus, dynamics of interest, and beam sensitivity.

In general, (S)TEM experiments can be broadly classified into two modes: parallel continuous-beam (TEM) and convergent scanning-beam (STEM). TEM mode collects continuous images of the specimen (i.e., by illuminating the region of interest all at once and collecting the transmitted signal on a 2D detector) with phase and diffraction contrast; this approach allows researchers to obtain information from the specimen at higher frames per second with a low electron dose rate, enabling high magnification and faster data collection rates for dynamic studies. The dose efficiency of TEM makes it popular for biological imaging and analysis of beam-sensitive specimens. In contrast, STEM mode uses a convergent probe that is serially scanned across the specimen; the scattered electrons are then collected, usually on an annular single-pixel detector, yielding a variety of easily interpretable signals, including atomic number (Z) contrast images.^56^ Compared to TEM, STEM typically requires higher dose rates but also enables extremely detailed and easily interpretable imaging, which makes it popular for studies of hard matter. Additionally, many parameters (e.g., dwell time, beam current, probe and pixel size etc.) affect the specimen damage threshold, and recent work^57,58^ suggests that damage thresholds in STEM may be lowered by tuning a number of acquisition parameters not considered in common damage tests for TEM mode (e.g., order of scan points within the scanning area^59^ and percentage of scan points^60^). For these reasons, a strict merit comparison between STEM and TEM for beam-sensitive materials is complex. Conveniently, STEM pairs well with additional detectors (e.g., acquiring simultaneous spatially resolved spectroscopic signals via EDS and EELS) and recent pixelated detectors can even be used to collect a 2D diffraction pattern at each scan position, resulting in so-called “4D-STEM” data sets.^61^ However, STEM is less ideal for studying rapid dynamic processes, because the rate of the observable material dynamics may exceed the maximum achievable frame rates (i.e., the dynamics may occur faster than the STEM can scan), resulting in distorted images.

Regardless of the mode and detectors being utilized, it is crucial to identify the effects of the beam on the environment and to choose beam parameters that minimize or eliminate those effects. Some data streams come at no “cost” (i.e., do not require a higher electron dose or increase risk of damaging a sample during characterization), including the use of multiple STEM detectors with different collection angles. However, other data streams (e.g., EDS) require additional dose to ensure sufficient signal-to-noise ratio (SNR). Similarly, EELS often requires slower data acquisition rates, which may limit the useful material dynamics information that can be captured. To limit the cost, it may be possible to keep the acquisition rate high and strategically bin the data in postprocessing to acquire high enough SNR for analysis.

### Recognizing Beam Effects

For in situ experiments, the specimen is accurately represented when the observations are reproducible and free of artifacts. Here, we explore the origin, identification, and mitigation of artifacts produced by the electron beam.

Electron-beam-induced artifacts are a concern for both ex situ and in situ (S)TEM experiments, but are often more limiting for in situ experiments due to the cumulative electron dose required for time-series acquisitions and the potentially damaging beam interaction with the applied environment. Common artifacts that have been reported over the years are^62^ 1) *structural damage*, which is typically observed as a smearing of atomic column contrast in high-resolution images, a fading of spots or rings in electron diffraction patterns, or a loss of spectral fine structure in EELS; 2) *mass loss*, which occurs when incident electrons displace atoms, thinning the specimen; 3) *specimen motion*, which blurs images and prevents high-resolution acquisitions; 4) *charging effects*, which are typically observed as image distortions in nonconductive specimens and can also prevent high-resolution acquisitions; 5) *chemical reactions with the environment*, which occur when the environment (e.g., liquid/gas) causes contamination build-up, even in high vacuum conditions; and 6) *growth of nanoparticles in the viewing area*, which can be caused by either chemical reactions with the environment or structural damage followed by longer range atomic motion (local mass loss in one area and eventual growth or agglomeration elsewhere).

### Identifying Beam Damage Mechanisms

For in situ (S)TEM, it is important to assess beam-sensitive materials to determine the dose-rate threshold or maximum dose that does not cause observable damage (e.g., morphological changes, mass loss, loss of EELS fine structure, solution degradation, or particle growth).^63^ Having said that, “observable damage” is a practical but somewhat misleading threshold because local changes to chemistry, particularly due to radiolysis damage (see below), will inevitably occur during characterization, even if the changes are insufficient to trigger an observable effect.

Fortunately, the artifacts induced by the electron beam during in situ experiments can be minimized, or even avoided, if appropriate countermeasures are taken (and we will discuss this in the next section). However, to establish effective mitigation procedures for specific beam-induced artifacts, it is necessary to first understand how beam damage comes about. As incident electrons pass through a specimen, they undergo elastic and inelastic scattering. These interactions are not only the source of imaging and spectroscopic signals but also potential sources of damage. The most common beam damage mechanisms in (S)TEM are knock-on displacement and radiolysis. *Knock-on displacement* occurs due to elastic scattering when the incident electron energy exceeds a material/element-specific threshold. It proceeds by transfer of a certain amount of kinetic energy to the specimen that is sufficient to “knock” an atom out of position. Characteristics of knock-on displacement that can help identify it as a main damage mechanism are 1) it does not vary much with temperature, and thus it can be considered independent of temperature; and 2) a primary/incident energy threshold, E_0_, exists below which knock-on damage does not occur.^62^ Therefore, we can evaluate and counter knock-on displacement by reducing the operating voltage and observing whether the effect slows or is eliminated. In situ and *operando* experiments focused on metals or semiconductors (e.g., in situ mechanical testing of metallic nanomaterials) are among those most likely to be primarily affected by knock-on displacement. *Radiolysis* occurs due to inelastic scattering when incident electrons break bonds and form different chemical species. Radiolysis can severely impact many in situ experiments (e.g., those involving liquid/gas environments, organic materials, or biological materials). Characteristics of radiolysis damage that can help identify it as a main beam damage mechanism are 1) it depends strongly on temperature (a reduction of the effects of radiolysis is expected by cooling the specimen); and 2) damage by radiolysis will increase for decreasing primary electron energy. We also note that inelastic scattering can cause other observable effects, such as charging or local heating. Charging effects are caused by emission of secondary electrons and Auger electrons, and are, thus, more likely to be observed in poorly conducting specimens.

A quantity named critical or characteristic dose can be estimated for a specific material and is defined as the dose at which some observable feature (e.g., a diffraction spot or energy-loss peak) decreases in intensity by a factor of Euler’s number. References showing determination of damage sensitivity of different systems using different electron microscopy techniques are available.^62,64^ The evaluation of critical doses for damage provides us with quantities of the total dose that we can allocate or distribute over multiple acquisitions and a range of possible operating conditions to improve the reliability of results.

Because in situ and *operando* experiments with gaseous or liquid environments are particularly vulnerable to radiolysis, it is also important to note that radiolysis can have different effects based on the state of matter (gases, liquids, solids). The initial radiolysis processes (i.e., energy deposition followed by fast relaxation processes) are independent of whether a material is in the gas, liquid, or solid state.^65,66^ For example, in the (S)TEM, all water molecules (water vapor, liquid water, and ice molecules) undergo radiolysis at about 1 fs, which triggers the formation of ionized water molecules (H_2_O^+^), excited water molecules (H_2_O*), and electrons (e^–^). For a brief time interval, these early radiolytic products (ions, excited molecules, electrons) tend to be heterogeneously distributed, regardless of phase. However, at longer time intervals, the final products of radiolysis will be different for different physical states.

The observable effects of radiolysis in liquid/solid phase experiments will tend to be driven by the radicals and molecular species produced.^67−69^ Due to a higher density of molecules in a liquid/solid, the initial ions and excited molecules will be closer together than in the gas phase. Therefore, these species and any radicals derived from them will react among themselves to some extent before diffusion.

In contrast, the observable effects of radiolysis in the gas phase will tend to be driven more so by ionization-based effects as compared to those in liquids. Indeed, ionization of gas molecules and its effects near/at the specimen surface have been consistently reported in gas phase in situ (S)TEM experiments.^70^ Typically, early radiolytic products (ions, excited molecules, radicals) formed in a material are heterogeneously distributed, and there is a competition between recombination to form molecular species and diffusion into the bulk. In gases, early radiolytic products will tend to diffuse away from the irradiated area, due to the much lower density of molecules, and reach an homogeneous distribution. The lifetime of the products formed in this case can thus be relatively long. We note that, if the lifetime of the products formed is shorter than the time between collisions, then little recombination will be expected in the gas. In practice, this results in a higher concentration of lower molecular weight products^71^ as well as more “surface” or “wall effects” as compare to the liquid phase.^72−74^ We note that most radiation chemistry work has been devoted to liquid samples and the radiation chemistry of gases is less well understood.

To provide a more detailed, practical example, Figure 4 shows an incident electron beam affecting a specimen during an in situ electrochemical experiment, including induced image artifacts and affected property measurements. Specifically, when the electron beam hit the electrode, it changed the measured voltage profile because the current being supplied to the electrode by the galvanostat (20 pA) was augmented by additional current supplied by the electron beam (14.5 pA), generating small spikes in the voltage profile whenever the electron beam scanned over the electrode. The electron beam also inflicted radiolytic damage on both the electrolyte and deposited lithium nuclei, which impacted the electrodeposition of lithium on the electrode surface, changing the morphology from high aspect ratio grains to rounded smaller grains. The beam’s influence on this site-specific measurement was easily captured because the researchers equipped the electrochemical cell with a 0.26 μm^2^ custom-patterned active electrode, ensuring that the voltage profile could be obtained from the same area that was resolved during imaging, to more clearly enable a holistic analysis of how imaging frequency impacts property measurements and highlight the importance of carefully assessing beam effects on both (S)TEM data acquisitions and property measurements during in situ and *operando* experiments.^22^

### Managing Beam Effects during In Situ Experiments

In general, the common techniques used to minimize beam damage during ex situ (S)TEM experiments are also effective for minimizing beam damage during in situ and *operando* (S)TEM experiments. However, additional methods specifically designed for in situ and *operando* experiments have been (and are continually being) developed.

#### Reduced Operating Voltage

For materials affected by knock-on damage, reducing the incident beam energy can minimize damage or even eliminate it (if observations can be performed below the knock-on threshold). For example, several metallic nanomaterials that are commonly studied with mechanical testing (e.g., Ti, Cu, Ni, Co, Nb or Au) have a primary energy threshold >300 kV;^75−77^ however, the primary energy threshold is lower for metallic specimens with low or medium atomic numbers, such as aluminum (180 kV)^75,76^ or graphite (150 kV).^78^ Lower primary energy thresholds are challenging because reducing the incident energy to reduce knock-on damage can increase the damage caused by radiolysis (which generally increases as the specimen’s primary energy decreases), as reported for specific 2D materials.^79^ In practice, most materials suffering primarily from knock-on damage will display much lower overall damage if experiments are performed near or right below the knock-on threshold.

#### Specimen Coating

Knock-on and charging effects can be reduced by the application of a specimen coating that acts as both a diffusion barrier (limiting the sputtering of species) and a conductive layers (preventing charging).^62^ However, we also note that the use of conductive coatings in in situ and *operando* experiments can produce two detrimental secondary effects. First, an increase in overall specimen thickness will produce additional inelastic scattering, which can result in decreased image and spectral quality. Second, for specimens primarily affected by radiolysis (e.g., those studied in liquid cell experiments), changes in chemistry due to the presence of interfaces can occur. For instance, additional inelastic scattering can result in local increased production of secondary electrons that transfer into the liquid. Even a thin metallic specimen coating could potentially change the chemistry of a solution if radiolysis occurs at the coating interface; this effect has been demonstrated for graphene coatings, which have been proposed to act as radical scavengers of damaging species such as hydroxyl radicals.^80^

#### Specimen Cooling

One of the most general methods for reducing (S)TEM beam damage is to cool specimens using cryogens (e.g., liquid nitrogen or helium). Researchers have observed that radiolysis strongly depends on temperature, and cooling has proven to be effective at reducing the diffusion of damaging radiolysis products. However, while cryofixation of samples in ex situ electron microscopy is an effective way to quench a process or maintain the state of a sample while minimizing radiolysis, it might prevent in situ or *operando* observations altogether.

#### Reduced Beam Exposure

The most straightforward way to control radiation damage is to reduce beam exposure, and a significant number of methods have been developed to achieve this. Conventional low-dose imaging uses software to find an area of interest using very low electron exposures, determine the appropriate focus in a nearby area, and finally deflect the electron beam to capture the area of interest at high resolution and with minimal pre-exposure. For many experiments, lowering the dose rate is more efficient than lowering the dose itself,^81−83^ particularly for experiments performed in gas environments.^82,83^ It is also possible to use dose fractionation or dose partition methods. In STEM-EELS, less beam damage has been observed in faster multiple scans acquired as compared to a slower single scan.^84^ Ultimately, for a given instrument configuration, reduced beam exposure will reduce specimen damage, but the achievable resolution of a material or process is ultimately limited by a specific material’s critical dose.

#### Signal Enhancement

To enable further reduction in beam exposure, researchers have implemented many methods to maximize the signal (i.e., enhance the information content for a given exposure): developing more efficient detectors, optimizing data collection strategies, using high-contrast enhancement methods (e.g., phase plates), and improving data processing to optimize the SNR obtained from limited beam exposure. Microscope settings and instrumentation can be optimized to increase sensitivity, but they must also be tuned for increased resolution, taking into account that resolution always depends on specimen thickness, the material’s intrinsic beam sensitivity, environmental factors (e.g., surface species, liquid/gas, temperature), and microscopy technique (spectroscopy requires much higher doses than TEM or STEM imaging, while diffraction techniques generally require lower doses).

#### Species Injection

A very different strategy to counteract radiolysis damage is to inject fresh species during observations. For example, in one in situ gas phase experiment, it appeared that the beam-induced reduction of ceria could be compensated by injecting oxygen in the gas environment during characterization.^85^ In the liquid phase, the concept of “flushing away” radiolytic species has been pursued for years, but recent modeling proves that it does not work in a straightforward way.^86^ This is currently an approach being explored, especially for its use in electrochemical studies.

In spite of our many mitigation strategies, there may still be some beam-induced effects that occur during in situ and *operando* (S)TEM experiments. To be fully cognizant of any beam effects, it is important to collect the right information from the specimen, both before and after the stimulus or bias is applied. In cases where the beam will have unavoidable effects on the specimen, it is best to collect initial-state data using low-dose conditions and to only expose small regions of the imaging area to the beam, ideally, preventing damage to the region of interest or neighboring regions prior to the in situ or *operando* experiment. In running the experiment, similar considerations persist. For example, in liquid cells, the region of the cell being imaged may display different reactions than the region outside of the beam; in mechanical experiments, imaging may induce local heating or elastic properties that are not evident in unilluminated areas. These examples demonstrate the importance of comparison studies of structural and nanoscale property measurements on the specimen, with and without the beam interaction. To capture “without beam” measurements, the researcher will collect an image at the initial state and run the same tests that were previously performed in situ or *operando* while the electron beam is blanked, then acquire a post-state image to compare with the initial-state data. Advances in low-dose detectors and software for precise beam control and exposure tracking have enabled better reproducibilitiy and control during in situ experiments. Further advancements in microscope control and automation will dramatically impact the reproducibility of these in situ and *operando* experiments, with enhanced software (to extract information from low signal-to-noise data sets) and programmable experiments (to reduce operator and collection errors), as described in more detail in the Data Analytics section below.

Section summary:

- The microscope’s alignment, imaging mode, beam parameters, and acquisition rates can determine the relative impact of beam effects observed during the experiment.
- The most common beam damage mechanisms in the (S)TEM are knock-on displacement and radiolysis.
- Mitigating the beam effects can be attempted through reducing the incident beam energy, coating the specimen, cooling the specimen to cryogenic temperatures, reducing beam exposure, enhancing the signal through advanced detectors or data processing methods, and injecting fresh species.

## Data Analytics: Setting Analysis in Motion

Meticulously designed and executed in situ and *operando* experiments can yield a wealth of high spatial, chemical, and temporal resolution data. However, the true power of these experiments lies in our ability to extract meaningful knowledge from the resulting data deluge.^87^ Therefore, it is important to approach experiment planning with the output data firmly in mind, both envisioning the data’s form and defining the exact analytical task that it will serve. A useful first step may be to translate materials science inquiries into the language of data science.^88^ For example, where a materials scientist might ask, “What is this crystal structure?” or “How fast does this phase transformation occur?,” a data scientist would rephrase these questions as, “Classify each image pixel into predefined categories” or “Fit a function describing the phase transition rate based on image/spectroscopic signals.” Being able to “code switch” between these linguistic registers will help a researcher pair their (S)TEM experiment design with the most appropriate statistical, artificial intelligence (AI), or machine learning (ML)-based analytical methodologies.

Conventional statistical analysis techniques offer a robust starting point for interpreting microscope data.^89,90^*Regression analysis methods* (e.g., fitting atomic-resolution images or spectroscopic peaks) are simple and easily interpretable because the fitted parameters often correspond directly to physical quantities. There are a variety of packages that can be used for this task, including ImageJ,^91^ HyperSpy,^92^ and Atomap.^93^*Hypothesis testing methods* (e.g., chi-squared tests, *t* tests, and ANOVA) can assess relationships between variables, such as comparing nanoparticle size distributions between different reactions. *Dimensionality reduction techniques* (e.g., principal component analysis, PCA) can help filter noise, enhance signal-to-noise ratios, and uncover correlations in large, multidimensional data sets.^94^ All of these conventional approaches are often readily implementable using existing code libraries, but have limitations when dealing with the increasingly high-dimensional, multimodal data generated by modern in situ experiments.^95^

**Figure 5:**
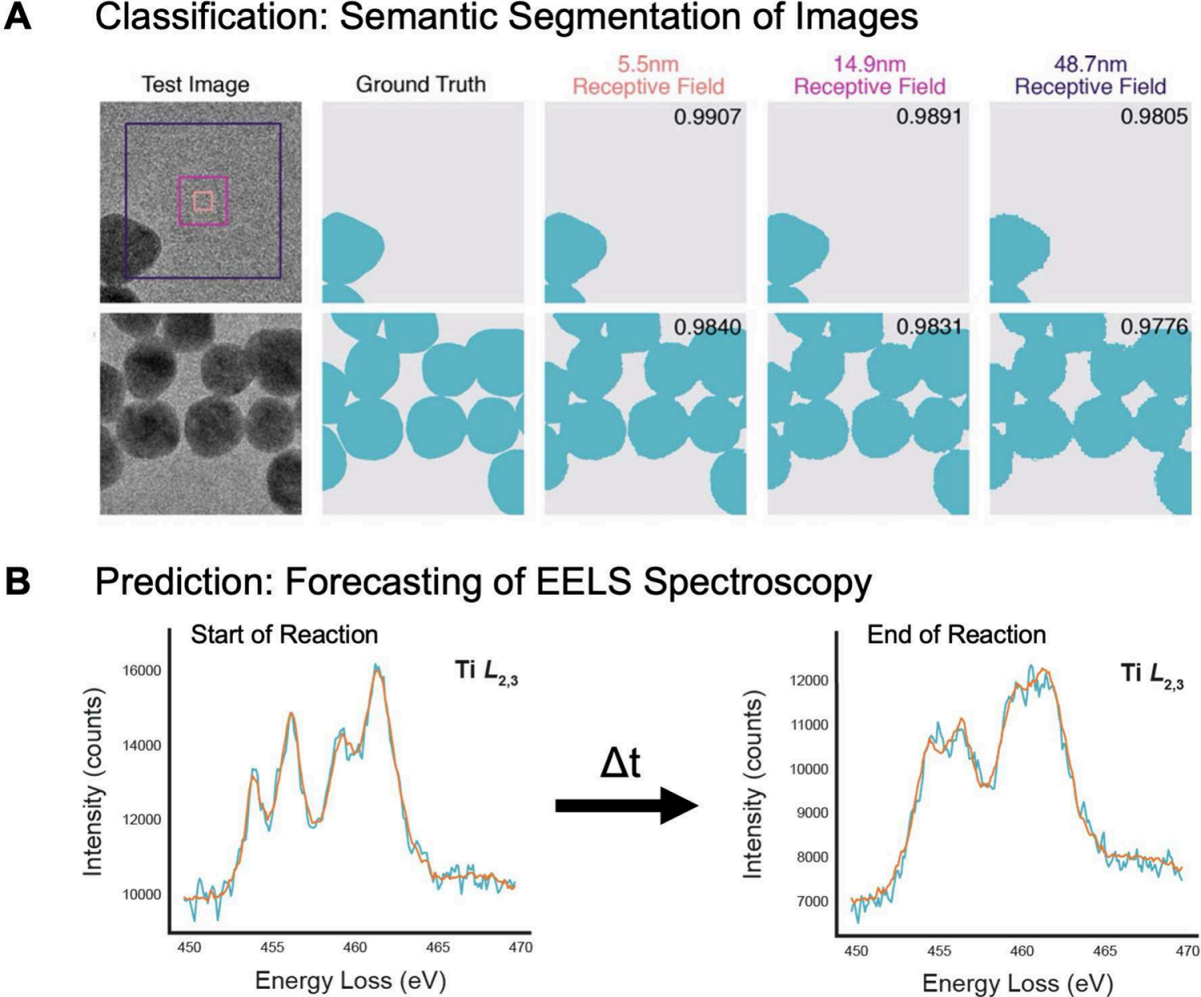
Machine learning interpretation of microscopy data. (A) ML identification of the size and distribution of a gold nanoparticle test image (column 1); (column 2) human-labeled ground truth data; (columns 3–5) the effect of receptive field size (i.e., area of the input image used by the neural network to generate the output) on segmentation. Adjusting these settings is key to getting accurate results. (B) ML prediction of how materials change over time, forecasting changes in the functional oxide SrTiO_3_ (as measured by EELS) as it undergoes a beam-induced reaction. Experimental spectra and model output are shown in blue and orange, respectively. Choosing the right amount of past data to use and how far into the future to predict are important for getting good predictions. A is adapted with permission under a CC BY license from Sytwu et al.^96^ Copyright 2022 Cambridge University Press. B is adapted with permission under a CC BY license from Lewis et al.^97^ Copyright 2022 Springer Nature.

The growing complexity of microscopy experiments has spurred the adoption of ML.^98,99^ Unlike simpler statistical models, which focus primarily on inference, ML models create predictions and generalizations that are better suited to more complex data and excel in the analysis of large data sets. As an example of the advantages of ML models, we consider the tasks of segmentation and forecasting shown in Figure 5; while these tasks could be conducted using simpler models, ML is more robust and offers greater predictive power. Moreover, as we enter an era of big data, ML tools can help to standardize in situ data collection and analysis, improve reproducibility, and uncover subtle mechanisms that might elude human observation. Early attempts to adapt ML methods for (S)TEM data analysis simply borrowed models from other domains, but it has quickly become evident that the specific characteristics of electron microscopy data (including the ability to acquire multiple signals concurrently) require specialized approaches.^87^ In situ and *operando* data is particularly challenging because it is inherently time-varying, multimodal, and relatively sparse.^100^ Thus, there are several critical considerations for ML in in situ and *operando* experiments:

- *Data Acquisition Timing:* The parallel or serial nature of image acquisition can introduce distortions, drift, scan errors, and/or radiation damage,^84^ so effective ML models must account for signal synchronization, noise, and potential artifacts. Benchmarking against ex situ measurements can help validate results and rule out artifacts.
- *Multimodal Data:* Most existing ML models are designed for unimodal data, but ML models that can leverage multiple data modalities could unlock enhanced descriptive power, facilitate data inpainting between modes, and improve physical interpretability.^101^
- *Sparse Data and Labels:* Despite the large volumes of data generated, the actual information content of (S)TEM data can be quite limited, due to factors like beam sensitivity or detector inefficiencies. Additionally, the scarcity or ambiguity of labels poses challenges for supervised ML approaches. Synthetic data generation or few/zero-shot learning techniques may offer potential solutions, but these approaches have both advantages and limitations.^102^

Complex data analytics tasks may require descriptive models capable of analyzing large, heterogeneous data sets with minimal human intervention. Deep learning has shown promise in tasks like particle and interface segmentation, especially when experimental variability is well-represented by simulations or prior examples.^103−105^ However, the broader application of deep learning in microscopy faces major analysis challenges, such as reconstructing STEM objects whose representation depends heavily on imaging conditions.^106^ In such cases, even sparse models that rely on limited examples may be valuable for triaging and selecting features of interest. Ultimately, the effective integration of ML into materials science research necessitates building collaboration between microscopy and data science that is driven by a deep understanding of the specific characteristics of microscopy data. By carefully considering factors like data acquisition timing, multimodality, and sparsity, are beginning to develop tailored models that unlock the full potential of these tools, which will enable valuable discoveries and deeper insights into the atomic world.

In an even more ambitious future, it may be possible step beyond ML data analytics into the domain of automated (and, potentially, autonomous) microscopy.^98,107,108^ Since its inception nearly a century ago, electron microscopy has been largely conducted by hand, with trained scientists curating, interpreting, and acting on data. Automation has the potential to disrupt this paradigm in many ways, with in situ microscopy standing to benefit greatly from advancements in calibration, reproducibility, and high-speed decision-making. Recently, instrument manufacturers have begun to develop sophisticated aberration-correction software and alignment routines to ensure consistent resolution; this standardized, automated calibration has enabled meaningful comparison of measurements across time and laboratories. However, standardization remains challenging for other experimental parameters, especially across custom-built or prototype systems.^109^ Emerging self-driving instrument platforms offer a potential solution. While scripting has been available for decades, the fragmented control architectures of many instruments have posed obstacles. However, emergent open-source instrument controllers and centralized ML-based platforms^107,110^ are enabling “open-loop” experiments (e.g., automated montaging and tilt series acquisition) that have predefined parameters executed consistently, enhancing reproducibility across sessions, users, and even laboratories. The most transformative potential lies in autonomous decision-making, or “closed-loop” experiments. Here, the system itself, guided by AI/ML models, makes decisions; the goal is to replicate human cognitive processes, harnessing the reproducibility, precision, and discovery power of such models to explore materials in greater depth. Predictive models (e.g., recurrent neural networks or transformers) coupled with Gaussian process optimization are particularly promising for real-time decision-making.^97,111,112^ While this field is complex, requiring centralized control, domain-specific models, and human validation, it promises to revolutionize in situ microscopy, elevating it beyond its current manual state. Continued research in this area will be essential to realizing its full potential.

Section summary:

- Intended analysis techniques should be considered during design of the experiment and data acquisition strategy.
- ML methods are good for advanced analysis tasks like segmentation and forecasting, particularly in noisy scenarios, but traditional statistical analysis are often more easily implemented and interpreted.
- In the future, careful implementation of autonomous approaches could enhance data acquisition, analysis, and decision-making during in situ experiments.

## Conclusions

This tutorial serves to set a baseline understanding of the critical considerations for planning and conducting in situ and *operando* (S)TEM experiments, which can be immensely powerful for understanding the nanoscale properties and dynamics of materials and reactions with the high spatial, temporal, and energy resolution required for site-specific investigations. However, the great value of these experiments is matched by their significant experimental complexity. At all stages of an in situ or *operando* experiment, researchers must think critically about what data they need, how they will acquire it, and how the acquisition could affect the materials or dynamics themselves. Fortunately, continuous advances in instrumentation and analysis tools are making reproducibility and reliability increasingly achievable, with automation poised to significantly reduce human error and improve efficiency of experiments. Broader access to advanced microscopes and holders is also allowing more researchers to gain experience and produce powerful results from in situ and *operando* methods. Together, (S)TEM technique advancement and growing capability access will steadily unlock more fundamental nanoscale property and mechanism insights over a wide range of different materials.
